# The potential carcinogenicity of orthopaedic implants – a scoping review

**DOI:** 10.1186/s12885-024-13279-2

**Published:** 2024-12-18

**Authors:** Cherry W.Y. Sun, Lawrence C.M. Lau, Jason P.Y. Cheung, Siu-Wai Choi

**Affiliations:** Department of Orthopaedics and Traumatology, School of Clinical Medicine, Faculty of Medicine, The University of Hong Kong, Hong Kong, China

**Keywords:** Orthopaedic implants, Malignancy, Implant metals, Carcinogenicity

## Abstract

**Background:**

Every year, hundreds of thousands of patients receive an orthopaedic or dental implant containing metals such as cobalt, chromium and titanium. Since the European Chemicals Agency (2020) classified pure cobalt metal as a Category 1B carcinogen, manufacturers of products containing ≥ 0.1% of this metal must perform a risk assessment and justify that there are no viable alternatives. The up-classification of cobalt metal to a carcinogen without good evidence that its use in implants is carcinogenic may cause unnecessary concern to the many patients who have, or may require such implants. Although in vitro and animal studies have shown such metals to be carcinogenic, human epidemiological studies have not been definitive. In addition, although many advances have been made in the past few decades with regard to the materials used in implant metals, no recent review of their carcinogenic effects have been published.

**Methods:**

This scoping review aims to summarise epidemiological studies conducted in recent years (from 2010 to present) to outline the carcinogenic effects of orthopaedic metal implants that have been published. This encompasses implants of different materials and surfaces, including metal, polyethylene and ceramic orthopaedic implants, cemented and cementless joint replacement surgeries, and surgical techniques such as resurfacing and total joint replacements that are currently in use and the potential carcinogenicity related to their use. Research papers with various study designs published in the English language were included. Studies were excluded if participants had a prior history of cancer before receiving orthopaedic implants and if they focused solely on the carcinogenicity of metals or materials not related to orthopaedic implants.

**Results:**

A total of 16 studies, encompassing over 700,000 implant patients, were identified through PubMed and have been included in this review. In long term follow-up of up to 17.9 years, no increased risk of all-site cancer was seen in these patients. However, an increase in site-specific cancers, namely prostate, melanoma and haematological cancers have been identified. Specifically, an increase in prostate cancer was identified in three studies.

**Conclusion:**

Based on the summarised evidence, there is no consistent evidence to show that patients with any type of orthopaedic implant has an increased risk of cancer, although slight (non-statistically significant) increases in prostate cancer was observed and this, in particular, deserves longer-term surveillance.

## Introduction

Orthopaedic implants play a vital role in modern medical procedures, serving as essential tools in the treatment and restoration of musculoskeletal functions. They are commonly used in procedures such as joint replacements, spinal fusions, and fracture fixations. These implants can be made from various materials, including metals, polymers, ceramics, or a combination of these [1]. Among these materials, metals have gained widespread popularity due to their high strength, durability, and biocompatibility. Chromium, cobalt, and titanium have revolutionized the field of orthopaedics. They are extensively utilized metals in orthopaedic implants, owing to their favourable mechanical properties, resistance to corrosion, superior wear resistance and biocompatibility [2].

However, concerns have been raised regarding the potential carcinogenicity of metals used in orthopaedic implants [3]. While orthopaedic implants have greatly improved patient outcomes, long-term exposure to metals may pose potential health risks. Assessment of the potential carcinogenicity of metals used in implants is a critical aspect that needs to be addressed to ensure patient safety. Understanding the potential carcinogenic effects of metals in orthopaedic implants can help clinicians make informed decisions, develop appropriate follow-up protocols, and mitigate any potential risks. Although these metals are generally considered biocompatible, there is emerging evidence suggesting that chronic exposure to certain metal ions released from implants may have carcinogenic and inflammatory effects. Chromium and cobalt, in particular, have been associated with adverse local and systemic reactions, including genotoxicity and mutagenicity [4, 5]. Titanium, on the other hand, has been considered relatively inert with high corrosion resistance, biocompatibility, repassivation, and adequate mechanical properties [6], although recent studies have raised concerns about its potential carcinogenic properties. In addition, increased plasma concentrations of titanium ions in patients with titanium implants have led investigators to question whether this metal is truly inert [7].

Although the number of patients requiring orthopaedic implants have increased worldwide in the last decade due to an ageing population, no current review of the safety of these implants have been conducted in recent years. We therefore sought to provide an update on studies published in the last ten years and aim to summarise the current understanding of the carcinogenicity of chromium, cobalt, and titanium in orthopaedic implants, to review their clinical significance and provide insights for future research, long-term implications and need for vigilance for patients with orthopaedic implants.

## Methods

### Search strategy

This scoping literature review conducted involved the detailed investigation of the carcinogenicity of chromium, cobalt and titanium in human epidemiological studies. Studies on animals and in vitro studies have not been included here. A systematic search was conducted in the PubMed database, with inclusion criteria; publication within the last 15 years (from 2010 to the present) with search terms including combination of keywords (e.g., “orthopaedic implants,” “metal implants”, “joint replacements,” “spinal fusions”, “fracture fixation”), the specific metals of interest (e.g., “chromium,” “cobalt,” “titanium”) and their significance (e.g., “carcinogenicity”, “genotoxicity”, “mutagenicity”, “metal ion release”). Boolean operators (“AND,” “OR”) were used to combine these MeSH terms effectively. A total of 108 articles were identified through PubMed. Although a similar search was conducted using the Cochrane Library and EMBASE, no further studies which were not in the original PubMed search were found.

### Inclusion and exclusion criteria

Only research papers published in the English language were included. Studies where full texts were available with various study designs (e.g., cohort studies, case-control studies) were considered. Specifically, studies examining both metal-on-metal and non-metal-on-metal implants were included to capture the full spectrum of implant types and their potential carcinogenicity. Studies which included participants with a pre-existing history of cancer before receiving orthopaedic implants were excluded, as were studies which only investigated the carcinogenicity of metals or materials unrelated to orthopaedic implants. No limit was placed on study sample size.

### Data extraction

**Fig. 1:**
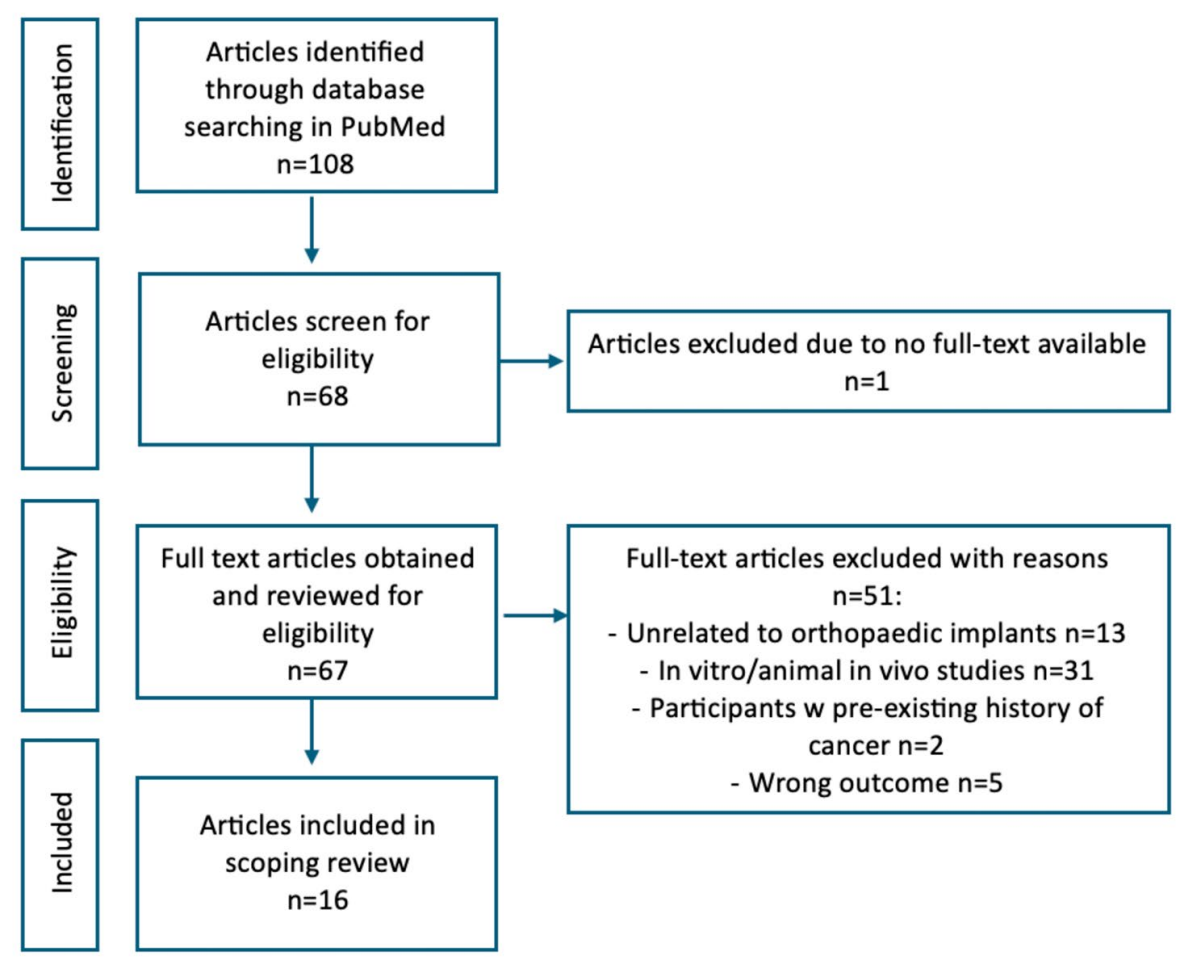
Flow diagram of literature search and study selection

Information including study characteristics (e.g., author, year of publication, study design), patient characteristics (e.g., sample size, demographics), implant characteristics (e.g., type of metal implant, duration of implantation), and outcomes related to carcinogenicity (e.g., incidence of cancer, cancer types) were extracted from each publication up till 22nd October 2024. CWYS and SWC constructed the fields in the data extraction table and this was confirmed by LCML and JPYC. CWYS conducted all the initial data extraction and this was further ascertained by SWC. Since this study is not a meta-analysis, the metrics used to assess randomised controlled trials were not applied in this instance, and all studies falling into the inclusion criteria have been included here (Fig. 1).

## Results

A total of 108 studies were identified through database searching in PubMed. After screening for eligibility (Fig. 1), a total of 16 studies are included in this review, encompassing various study designs, with follow-up periods ranging from 3 to 17.9 years (Table 1). The sample sizes varied across studies, with patients with total hip arthroplasty being the most common population studied. The most common metal in use was metal implants made of cobalt-chromium metal alloy. Overall, results suggest that there is no consistent evidence of an increased risk of cancer associated with metal-on-metal (MoM) hip implants.

**Table 1:** Summary of studies investigating the relationship between orthopaedic implants and subsequent malignancies

Study	Study design	Sample Size	Follow-up period	Metal implant type	Metal in use	Main outcome measure	Carcinogenicity outcome	Results
[8]	Cohort	24,636 patients with primary osteoarthritis	13 years	MoP THA	CoCrMo, TiAlV, SS alloy	Standardized incidence ratio (SIR) (numbers of cancer cases observed compared with expected rates based on incidence in the general population)	No increased cancer risk; Cancer SIR: 0.98 (CI: 0.94–1.03) ≥ 10 years follow-up	No all-site/site-specific cancer risk, mean follow-up, 12.6 years
[9]	Retrospective cohort	579 MoM vs. 1585 MoP	17.9 years (patients with MoM) 16.7 years (patients with MoP)	MoM and MoP THA	CoCr	Standardized mortality ratios (SMR) of total and site-specific causes of death	No increased cancer mortality rate in MoM vs. general population in mean follow-up time of 17.9 years. Higher standard mortality rate in MoM > MoP, RR of 1.36 (CI: 1.02–1.79) in first 20 years post-op. No practical difference ≥ 20 years Overall SMR was 0.95 for the MoM and 0.90 for the MoP cohort, compared to general population. Both cohorts showed significantly decreased mortality for the first decade postoperatively, equal mortality over the next 10 years, and significantly increased mortality after 20 years. Patients with MoM THA, higher cancer mortality (SMR 1.01) than those with MoP THA (SMR 0.66) during the first 20 years postoperatively, but not thereafter.	No increased cancer mortality rate in MoM THA
[10]	Retrospective Cohort	8343 patients with THAs	9 years	MoM and other bearings THA	Ti-alloy, CoCr-alloy, Fe-alloy	SIR and Poisson regression relative risks (RR) calculated for all and specific cancers	Risk of prostate cancer increase in MoM (SIR: 1.35), MoP (SIR: 1.30), non-cemented THA (SIR: 1.40); Titanium alloy THA (SIR: 1.41); overall prostate cancer risk RR: 2.02 Risk of skin cancer increased RR: 1.92)	Cancer risk not increased (SIR: 0.97; CI: 0.93–1.03)
[11]	Retrospective Cohort	71,990 patients with primary THA or primary resurfacing arthroplasty	7.6 years	MoM THA	Chromium, cobalt, nickel, aluminium alloys	SIR cancer in cohort group	Overall excess risk of cancer of 5%; Risk of basal cell carcinoma of skin increased (SIR: 1.11, CI: 1.04–1.18); multiple myeloma and other immunoproliferative neoplasms (SIR: 1.37; CI: 1.09–1.70); all cancers combined (SIR: 1.10; CI: 1.07–1.13) in 1–4 years post-op	Overall excess cancer risk seems unlikely to be of aetiological/ clinical significance; No statistically significant evidence of increased risk of cancer after ≥ 10 years follow-up
[12]	Cohort	91,291 patients with unilateral knee arthroplasty (UKA) or TKA	6 years	TKA	/	SIR cancer in cohort group	Overall cancer risk elevated by 10–26%; Increased risk of myelodysplastic syndrome, prostate cancer and melanoma likely contributed by prostheses metal exposure	Increased in all-site cancer risks; Increased risk of myelodysplastic syndrome, prostate cancer and melanoma
[13]	Retrospective Cohort	156,516 patients with conventional stemmed THA procedures and 11,321 with resurfacing THA procedures for osteoarthritis	5 years	MoM and non-MoM THA	/	SIR cancer in cohort group	All-cause cancer risk for csTHA procedures significantly higher (SIR 1.24, 95% CI 1.22–1.26). Site-specific cancer rates significantly higher for prostate cancer (SIR 1.46, 95% CI 1.41–1.51), melanoma (SIR 1.53, 95% CI 1.46–1.61), non-Hodgkin’s lymphoma (SIR 1.25, 95% CI 1.14–1.35), myeloma (SIR 1.60, 95% CI 1.41–1.79), leukaemia (SIR 1.21, 95% CI 1.10–1.33), colon (SIR 1.24, 95% CI 1.18–1.30), bladder (SIR 1.15, 95% CI 1.03–1.27) and kidney (SIR 1.22, 95% CI 1.09–1.35); Resurfacing THA procedures all-cause cancer risk significantly higher (SIR 1.42, 95% CI 1.32–1.51) Individual cancer sub-types risk significantly higher includes prostate cancer (SIR 2.30, 95% CI 2.05–2.55) and melanoma (SIR 1.72, 95% CI 1.39–2.04)	Overall cancer risk increased compared to general population, no increased cancer risk associated with specific bearing surfaces
[14]	Retrospective Cohort	40,576 (hip arthroplasty with MoM bearing surfaces) 248 995 (alternative bearings)	3 years	MoM and other bearings THA	Cobalt, chromium, molybdenum	Incidence of all cancers and incidence of malignant melanoma and prostate, renal tract, and haematological cancers	No link identified	No increased cancer risk with MoM bearing surfaces 7 years post-op
[15]	Cohort	403,881 patients with MoM THA	4.6 years	MoM THA	/	Incidence of new cancer diagnosis	No increased cancer risk	No increased cancer risk
[16]	Retrospective Cohort	10 728 patients with MoM THA and 18 235 patients with conventional non-MoM cohort (MoP, CoP, and CoC) THA	7.4 years in the MoM cohort and 8.4 years in the non-MoM cohort	MoM THA	/	SIR cancer in cohort and general population	No increased overall cancer risk (SIR: 0.9; CI: 0.9-1.0); Risk of basalioma increased (RR: 1.2 CI: 1.0-1.4)	No increased overall cancer risk in mean follow-up, 7.4 years
[17]	Cohort	10,728 MoM patients and 18,235 conventional THA	MoM (4.6 years) and non-MoM (6.0 years)	MoM and non-MoM THA	/	SIR cancer and SMR	Overall risk of cancer not increased; Risk of soft-tissue sarcoma and basalioma in MoM increased	Overall cancer risk not increased; RR of soft tissue sarcoma in MoM: 2.6 (CI: 1.0-6.4); RR of basalioma in MoM: 1.3 (CI: 1.1–1.5)
[18]	Retrospective Cohort	10,783 patients aged 50 years or over newly diagnosed with mature B-cell malignancy or precursor condition	/	Joint replacements	/	Odds ratio for cancer	MGUS and myeloma risks increased after both hip and knee arthroplasty (MGUS: OR = 1.2, 95% CI 1.0–1.5, *p* = 0.06 and OR = 1.5, 95% CI 1.2–1.8, *p* < 0.001; Myeloma: OR = 1.2, 95% CI 1.0–1.6, *p* = 0.08 and OR = 1.2, 95% CI 0.9–1.5, *p* = 0.24 for hip and knee, respectively); Hodgkin lymphoma risks increased with hip arthroplasty (OR = 1.9, 95% CI 1.0–3.4, *p* = 0.04)	Increased risk of MGUS and myeloma with THA and TKA
[49]	Cohort	11,540 patients with THA	3.4 years	MoM and other bearing surfaces THA	Cobalt, Chromium	Adjusted relative rates of cancer	No increased cancer risk	Not associated with increased cancer risk
[50]	Cohort	41,402 patients with THA	11.9 years	MoP THA	/	SIR cancer in the cohort	No increase in overall cancer risk (mainly MoP)	No increased in overall cancer risk
[51]	Retrospective Cohort	10 728 patients (MoM THA) and 18,235 patients (conventional non-MoM cohort (MoP, CoP, and CoC) THA)	4 years	MoM and other bearings THA	Chromium, Cobalt, Titanium	SIR cancer in the cohort group and general population	No increased overall cancer risk in mean follow- up of 4 years (RR: 0.92; CI: 0.81–1.05); Risk of soft tissue sarcoma increased but insignificant (RR: 2.69; CI: 0.89–6.71)	No increased overall cancer risk
[52]	Retrospective Cohort	9443 TKA patients	14 years	THA	/	SMR for total and site specific causes of death	Overall cancer mortality significantly reduced but showed increasing trend over time and reached mortality of normal population by 17th post-op year	No increased cancer risk in TKA
[55]	Retrospective Cohort	126,276 patients exposed to a cemented THA 555,757 unexposed individuals	14.6 years (exposed), 14.1 years (unexposed)group	THA	/	Cumulative cancer incidence, unadjusted and adjusted hazard ratios of cancer	Increased risk skin melanoma (HR: 1.15, CI: 1.05–1.24)	No increased overall cancer risk

With regard to all-site cancer incidence in patients with metal hip implants, thirteen studies reported no elevated cancer risk compared to the general population, with standardized incidence ratios (SIRs) ranging from 0.97 to 1.35 [8–11]. The average follow-up period for studies concluding no associations between cancer and total hip arthroplasty (THA) or total knee arthroplasty (TKA) is 8.88 years, which included orthopaedic metal implants made of various types of metals e.g. cobalt, chromium, titanium etc. A cohort study with a 13-year follow-up period on over 24,000 patients with metal-on-polyethylene (MoP) THA due to primary osteoarthritis found no increased cancer risk in patients with MoP THA compared to the general population [8]. Moreover, no increased cancer mortality rate was identified in patients with MoM or MoP THA when compared with the general population in a mean follow-up time of nearly 18 years [9]. Both the MoM and MoP cohorts showed significantly decreased cancer mortality in the first decade postoperatively, with equal cancer mortality compared to that of the general population over the next ten years. A slight increase in cancer mortality is noted in patients with MoM THA than those with MoP THA during the first two decades postoperatively, but not thereafter. Brewster et al. identified an overall excess risk of cancer of 5% in patients with MoM THA but no statistically significant evidence of increased risk of cancer after more than ten years of follow-up [11]. It is suggested that the overall, slight, excess cancer risk seems unlikely to be of aetiological or clinical significance. Some studies have even identified that patients with THA have a superior 10-year life expectancy than that of the general population [9, 11]. Yet, they attributed that observed increased life expectancy to the ‘healthy patient effect’, where patients tolerating major surgeries such as THA or TKA have better overall fitness.

In comparison, two studies, a Swedish cohort study on patients with knee arthroplasties and an Australian nationwide linked registry cohort analysis found an increased all-site cancer incidence in patients with THA. Wagner et al. (2011) conducted a study with a median follow-up period of 6.05 years and reported a significantly elevated overall cancer risk in the study cohorts with an excess cancer incidence of 10–26% compared to the general population [12]. Pratt et al. (2022) also showed a significant increase in all-cause cancer incidence in patients with THA compared to the general population with an analysis involving over 167,000 patients, where patients with conventional stemmed THA exhibited an overall 24% greater risk of developing cancer of any kind compared to individuals in the Australian population who share similar age and gender distribution [13].

For site-specific cancer incidence, six studies did not observe any increased specific cancer incidence in the study population. Smith et al. (2012) stated no association was discovered between MoM THA regardless of whether they were stemmed or resurfacing, and an elevated risk of cancer diagnosis [14]. The occurrence of cancer diagnosis following hip replacement is infrequent and lower than the expected incidence in individuals of the same age and sex in the general population. Specifically, patients with resurfacing THA were revealed to have a reduced likelihood of prostate cancer and haematological cancer diagnosis. Moreover, a research extended the duration of follow-up compared to previous studies and conclusively demonstrated no heightened risk of developing site-specific cancers, including haematological cancer, malignant melanoma, cancer of the urinary tract, or prostate cancer in men, after undergoing primary hip replacement with a specific bearing surface material [15].

Conversely, eight studies noted a statistically significant increase in site-specific cancer incidences, with prostate cancer, melanoma and haematological or immunoproliferative neoplasms frequently mentioned. A review of the studies revealed mixed findings regarding site-specific cancer incidences. Although some studies did not observe an increase in all-site cancer incidence, an increase in site-specific cancer incidence in patients with metal implants compared to the general population has been identified. Increased cancer incidences in prostate cancer, skin cancers including melanoma and haematological cancers were sites frequently identified. Among all cancers with an increase in site-specific incidences in patients with metal implants, site-specific cancer incidence of prostate cancer yielded the highest relative risk and SIR. Three studies found a statistically significant increase in cancer incidence in prostate cancer in the study population with metal implants, with SIR ranging from 2.02 to 2.30 [10, 12, 13]. An increase in site-specific cancer rates of skin cancer including melanoma was noted in six studies, with SIR ranging from 1.11 to 1.92 [10–13, 16, 17]. Four studies identified an increased incidence of haematological or immunoproliferative neoplasms, when compared to the general population, with SIR ranging from 1.21 to 1.60 [11–13, 18]. In particular, myeloma risks were commonly identified to be statistically significantly increased in patients with THA.

No significant difference in all-site and site-specific cancer incidence was demonstrated in the studies associated with the difference in bearing surfaces in THA. Regarding mortality, studies indicated that MoM THA did not lead to increased cancer mortality compared to the general population. However, there were variations in mortality rates between different bearing surfaces and follow-up periods. Visuri et al. reported that patients with MoM THA had higher cancer mortality (SMR 1.01) than those with MoP THA (SMR 0.66) during the first 20 years postoperatively, but not thereafter [9].

## Discussion

Cobalt, chromium, and titanium are metals commonly used in orthopaedic implants due to their favourable mechanical properties and biocompatibility [19]. These metals play a crucial role in the construction of joint replacements, spinal fusions, and other orthopaedic devices.

Cobalt is a transition metal that exhibits high strength, corrosion and wear resistance, making it suitable for use in orthopaedic implants. The use of chromium in orthopaedics primarily involves its incorporation into cobalt-chromium (CoCr) alloys [20], and such alloys have been widely utilized in hip and knee replacements, as well as dental and orthopaedic screws. These alloys provide excellent mechanical stability, reducing the risk of implant failure. Cobalt-chromium implants have low friction properties, which contribute to improved joint mobility and longevity. Generally, CoCr hip or knee replacements are expected to last for 15–20 years or more. Hence, it is commonly used as bearing surfaces in metal on polyethylene (MoP) bearing applications in joint replacements as well as metal-on-metal bearings [21]. However, there is an increased concern regarding the release of cobalt and chromium ions from metal on metal (MoM) implants and that has been associated with adverse local tissue reactions and potential systemic effects. It was noticed that metal ions released from implants can recruit and activate osteoclast precursor cells while inhibiting osteoblasts, both of which are crucial in the development of periprosthetic osteolysis and implant loosening [22]. Other cobalt-associated systemic effects including neuro-ocular toxicity, cardiotoxicity and thyroid toxicity were observed in patients with elevated blood cobalt and chromium concentrations post-arthroplasty [23].

Titanium is a biocompatible metal extensively used in orthopaedic implants due to its excellent corrosion resistance and high strength-to-weight ratio. It is known to have the highest corrosion resistance among the commonly used metals (cobalt-chromium alloys, stainless steel alloys), with strength equivalent to steel while being 45% lighter and elasticity closely resembles that of healthy bone rather than steel [1, 24]. Titanium and its alloys, such as titanium-6 aluminum-4 vanadium (Ti-6Al-4 V), are commonly employed in joint replacements, spinal fusions, and bone plates [25]. Titanium implants have demonstrated good clinical outcomes and long-term survival rates. They promote osseointegration, the direct integration of the implant with the surrounding bone tissue by recruiting osteoblasts and allowing these cells to mature and differentiate. This enhances implant stability and reduces the risk of implant loosening [26]. Although titanium is comparatively more bioinert than CoCr and has a unique property of limiting implant loosening, the release of titanium particles and ions from implants can still occur due to wear, development of a bacterial biofilm, corrosion, and other mechanical factors. These released particles and ions have been shown to induce immune responses and inflammatory reactions in surrounding tissues [27]. When metal ions from orthopaedic implant wear debris bind to serum proteins, they result in hapten-like complexes that the immune system can recognise as antigens and potentially trigger an immune response. The production of pro-inflammatory cytokines was monitored in patients with aseptic loosening of THA, revealing significantly increased levels of IL-1β, IL-2, IL-8, IFN-γ and TNF-α [28]. Additionally, histological samples of patients after MoM THA exhibited lymphocytic infiltrate, indicating a Type IV delayed hypersensitivity reaction, where osteolysis and significant damage to the soft tissues were also observed [29].

While cobalt, chromium, and titanium are widely used in orthopaedic implants, concerns have emerged regarding their potential carcinogenicity. Several studies have investigated the release of metal ions from these implants and their potential systemic distribution, leading to tissue damage and potential carcinogenic effects. Preoperative and postoperative blood samples were collected to analyse the serum ion levels of aluminium, chromium, cobalt, and silver, reflecting postoperative median serum concentrations of chromium and cobalt increased by 11-fold and 64-fold respectively hence demonstrating the systemic distribution of metal ions [30]. Besides, peri-prosthetic soft tissue biopsies exhibited extensive necrosis, often penetrating more than 1 cm deep, accompanied by the presence of chromium particles and a high score of aseptic lymphocytic vasculitis-associated lesions (ALVALs) [31].

In particular, MoM hip implants have been extensively studied due to the higher likelihood of metal ion release compared to non-metal-on-metal implants. The wear and corrosion of the metal components in MoM hip implants can lead to the release of cobalt and chromium ions into the surrounding tissues and bloodstream. Elevated levels of these metal ions have been detected in the blood and urine of patients with MoM hip implants [32–34]. The release of cobalt and chromium ions from MoM hip implants has been associated with adverse local tissue reactions, such as metallosis, pseudotumors, and aseptic loosening [35]. These reactions can lead to pain, implant failure, and the need for revision surgery. In particular, aseptic failure is the failure of prosthetic component fixation in the absence of infection [36]. It is proposed that aseptic loosening is secondary to wear particles mediated inflammatory osteolysis. Wear debris is generated at various locations in prosthetic joints, including articulations, modular interfaces, and non-articulating interfaces. Phagocytosis of wear debris by macrophages is suggested to stimulate the secretion of proinflammatory factors, gelatinases and proteases, contributing to periprosthetic osteolysis [37]. The induced inflammatory response disrupts the homeostatic balance of osteoblastic and osteoclastic activities, with the potential involvement of the Wnt signalling pathway in wear particles associated aseptic loosening [38]. Associations between metal ions and wear debris with aseptic loosening is further validated by the presence of significantly higher concentrations of wear debris in regions with increased lysis.

Furthermore, systemic effects have been reported, including cardiotoxicity, neurotoxicity, and genotoxicity. An in vivo study on nematodes demonstrated that metal ions increase the cardiotoxicity associated with immunoglobulin light chain (LC) amyloidosis as they were implicated in the generation of reactive oxygen species and the subsequent oxidative stress and mitochondrial damage observed in cardiac tissues affected by LC amyloidosis [39]. Notably, cobalt ions exhibit preferential deposition in the myocardium, augmenting the likelihood of metal ion-induced cardiotoxicity [40]. Neurotoxicity due to metal ions, especially cobalt, can be explained by its ability to disrupt mitochondrial metabolism, which ultimately results in cellular dysfunction and cell death [41–43]. Studies have also shown an association between elevated cobalt and chromium levels and cellular toxicity, DNA damage, and potential mutagenic effects [44, 45]. Although there is in vitro and animal in vivo evidence that cobalt ions can interfere with DNA repair mechanisms and cause direct DNA damage and their potential carcinogenic effects, human studies are limited to occupational exposure of hard metal workers to cobalt dust and focus on the subsequent development of lung cancer.

As concerns regarding the risks associated with MoM THA have grown, the use of these implants has been declining. Following the identification of significantly higher association of uncemented MoM hip replacements with adverse soft tissue reaction when compared to other hip replacement material used, the Medicine and Healthcare Products Regulatory Agency (MHRA) issued a warning for MoM implants. Subsequently, the use of MoM hip replacements as uncemented primary hip replacement bearing surfaces has gradually declined since 2007 and has been almost completely out of market since 2012. The National Joint Registry 20th Annual Report in 2023 documented that MoM hip replacements is only less than 0.1% of all primary hip replacements from 2003 to 2023, with the most commonly used implant material continues to be cemented MoP, accounting for 85.3% of all cemented primary procedures [46].

### Epidemiological studies on carcinogenicity of orthopaedics metal implants

Despite the reported carcinogenicity of cobalt, chromium and titanium illustrated by multiple in vitro and animal studies, the majority of epidemiological studies conducted in patients with such implants suggest otherwise. Although a long-term follow-up study of THA with MoM bearings conducted in Italy identified significantly higher blood and urinary cobalt metal ion levels in the MoM group compared with Ceramic-on-Ceramic (CoC) group 7 years after surgery, none of the patients in MoM group exceeded the metal ion levels associated with metallosis [47]. They also evaluated the potential systemic effects of systemic metal debris dissemination with thyroid, liver and heart functionality tests but none of the patients with elevated metal ions present with altered organ functionality. A study in 2010 identified no increase in any all-site or site-specific cancer risk in patients with conventional THA made of CoCr and Ti-alloys [8]. There is no difference in the standardized incidence rate of cancer in the THA group and the general population within the long-term follow-up period. The disparity seen in the in vitro/animal studies and epidemiological human studies may be due to the differences between the metal ions used in in vitro studies to emulate the metal ions released from real-life implants in humans. Ions used in animal studies are often inorganic metal salts, while ions released into the human body from implants may be bonded to plasma proteins, conferring to these ions a different chemistry.

Similar results are reflected in a retrospective cohort study of patients with CoCr alloy hip prostheses, where it was demonstrated that there is no increased cancer mortality in the MoM group as compared to that of the general population, while conversely, the cancer mortality rate in patients with MoP hip prostheses was significantly reduced [9]. Comparing the standardized mortality rate between the MoM and MoP groups demonstrated that there is higher cancer mortality in patients with MoM THA compared to the MoP THA group in the first 20 years postoperatively, with no practical difference thereafter. Levašič et al. also found that general cancer risk in patients with THA is comparable to the general population but with the exception of a slight increase in prostate cancer risk [10]. Specifically, all-site cancer risk is higher in MoM cohorts than non-MoM, particularly, higher prostate and skin cancer risk has been identified in MoM compared to the non-MoM cohort. The increase in cancer mortality in MoM THA might be explained by the difference in serum metal ion levels found in MoM and MoP patients. In this regard, Dahlstrand et al. (2017) reported an increase of 2–4 folds in mean serum cobalt and chromium concentration in MoM compared to that of MoP [48]. Although there is no statistically significant difference in the implant survival of MoM and MoP, with the absence of clinical and physiological superiority of MoM bearing and long-term systemic elevation of metal ion levels with potential carcinogenicity, MoP should be the implant of choice to lessen the risk of increased serum concentrations of metal ions.

To investigate the differences in cancer mortality in patients with MoM THA compared to MoP THA, investigators have compared the cancer incidences in patients with MoM THA, THA with other bearing surfaces and the general population. Many cohort studies have in fact found the cancer incidence of MoM and other bearings THA was similar or even lower than the standardized cancer incidence rate of the normal population [14–16, 49]. When compared with alternative bearings, no increased risk of any cancer diagnosis or increased cancer mortality has been identified in the MoM patient group. Despite theoretical risks, there was no direct association between types of bearing surfaces and cancer risks. This finding is in accordance with the results of other similar studies. A prospective cohort study in Norway investigated a confounding factor, cemented or cementless implants, and its effect on carcinogenicity [50]. This study found no difference in cancer risk when comparing patients with THA to the general population. Although a slightly increased risk of cancer is identified in the group with uncemented THA than that of the patients with cemented THA, investigators proposed that it may be prone to unmeasured confounding factors, such as genetic predispositions, occupational exposure, other cancer risk factors including smoking and comorbidities. Individual studies have however identified a slight increase in the incidence of soft tissue sarcoma and basal cell carcinoma [17, 51], but the increased risk is insignificant, and not high enough to draw the conclusion that it is associated with THA. Brewster et al. identified an overall excess cancer risk of 5% in patients with THA, with a modestly increased risk of all cancers, prostate cancer and multiple myeloma, but again, there were confounding factors and no causation could be attributed to THA [11]. No increased cancer risks and cancer mortality rate identified in patients with total knee arthroplasty were found when mixed metals used in implants were considered [52].

With a substantial transition in the selection of materials for THA from MoM to alternative bearing surfaces, there has been a corresponding increase in research aimed at elucidating the differences in adverse effects and cancer incidence associated with these alternative materials. Brian et al. (2016) conducted a serological analysis of 80 patients with functional unilateral THA with four different bearing surfaces, including CoC, Ceramic-on-Polyethylene (CoP), MoP and dual mobility (DM) [53]. They did not identify significant differences in serum cobalt and chromium levels across the four bearing surface groups 1 year post-THA. Despite a higher serum cobalt levels is demonstrated in the MoP patient cohort, there was no correlation between metal ion levels and patient-reported outcome scores and showed little clinical significance. Yet, this study assumed that metal ion levels would plateau after one year and was limited by a follow-up period of only one year. Consequently, it is not possible to ascertain the long-term systemic effects and potential carcinogenicity of the elevated metal ion levels detected in the MoP group compared to the ceramic group. A case report of osteosarcoma around a CoC THA suggested that although CoC implants have the lowest wear rate compared to ceramic-on-metal and CoP implants, ceramic debris may contribute to adverse local tissue reactions with associated local tissue inflammation [54]. Nonetheless, it emphasised that sarcoma after THA is a rare occurrence and most pseudotumours have been identified as benign periprosthetic masses and non-cancerous in nature that it remains unclear whether the occurrence of periprosthetic osteosarcoma in CoC THA in this patient is coincidental. In most cases, there is insufficient and inadequate evidence to support the potential carcinogenicity of various orthopaedics implant materials used in total hip arthroplasty.

In contrast to studies which found negligible or no increased cancer risk, a study in patients on the Swedish Knee Arthroplasty Register showed contrary results. Analyses of this data showed an increased overall risk of cancer and risk of several individual cancer types compared with the general population, and inferred that myelodysplastic syndrome and possibly melanoma and prostate cancer could be associated with metal ion exposure from the implant [12]. There was an excess cancer incidence of 10–26% compared to the general population and among different cancer types, while myelodysplastic syndrome showed an increase of 3–5 folds in all subgroups. A report from the Haematological Malignancy Research Network verified the increased odds of haematological cancer, specifically mature B-cell neoplasms, after joint replacement [18]. It was found that the risk of Monoclonal Gammopathy of Unknown Significance (MGUS) and classical Hodgkin Lymphoma diagnosis was increased several years after joint replacements. A nationwide Australian cohort analysis of more than 167,000 patients also found a significantly higher all-cause cancer incidence in patients with conventional stemmed or resurfacing THA compared to the general population [13]. Site-specific cancer incidences were significantly higher in prostate cancer (SIR 1.46–2.30) and melanoma (1.53–1.72) for both conventional stemmed and resurfacing THA, and incidences of cancer were increased regardless of the bearing surfaces involved. A statistically significant risk of melanoma (HR 1.15; CI 1.05–1.24) was also detected in a nationwide cohort study in Sweden [55]. However, no overall increased cancer risk was found in the same study.

### Limitations to this review

This current scoping review is a collation of information from studies investigating the association between cancer risk and orthopaedic implants in human patients published in the past decade. It is limited by the studies published which are retrospective in nature and based on orthopaedic implant registry data which do not include information specific to the types of metals used in the implants. Therefore, it was not possible to identify if a specific metal is responsible for the slight increases in cancer risk seen in some studies. In the same vein, it was also not possible to arrange this review into separate sections regarding which metals have been identified to increase cancer risk. Nevertheless, the strength of this review is that it has encompassed information on all studies published in the last decade, has identified that MoM implants has been found to increase cancer risk in some studies, and has confirmed that MoP is a better choice in terms of the negligible increased cancer risk found in patients with MoP implants.

## Conclusion

In conclusion, this scoping review highlighted the importance of considering the duration of follow-up when assessing cancer risk in patients with orthopaedic implants. Latency is an important consideration in determining the cause of cancer and it is suggested here that early excess cancer risk during follow-up may be due to detection bias or disease-related aetiological factors. Some studies reported no increased cancer risk in the short term (e.g., 3–4 years post-op) [51], while others found potential risks emerging after longer follow-up periods. Therefore, long-term monitoring and further research are necessary to fully understand the potential carcinogenicity of MoM hip implants.

Based on the most recently available evidence, this review suggests that there is no consistent indication of an increased risk of cancer associated with implant materials in THA, particularly MoM hip arthroplasty. While some studies reported elevated risks for certain cancer types, overall, the findings do not support a definitive link between orthopaedic metal implants and carcinogenicity. However, the review emphasizes the need for continued monitoring and long-term, prospective studies to further evaluate the potential risks of orthopaedic metal implants, particularly those containing cobalt-chromium metal alloys.

## Data Availability

No datasets were generated or analysed during the current study.

## Abbreviations

MoM: Metal-on-metal
SIR: Standardized incidence ratio
SMR: Standardized mortality ratios
THA: Total hip arthroplasty
TKA: Total knee arthroplasty
MoP: Metal-on-polyethylene
CoCr: Cobalt-chromium
CoC: Ceramic-on-ceramic
CoP: Ceramic-on-polyethylene
DM: Dual mobility
ALVALs: Aseptic lymphocytic vasculitis-associated lesions
LC: Light chain
MGUS: Monoclonal gammopathy of unknown significance
